# Mass Spectrometric Immunoassay for the qualitative and quantitative analysis of the cytokine Macrophage Migration Inhibitory Factor (MIF)

**DOI:** 10.1186/s12953-014-0052-3

**Published:** 2014-10-14

**Authors:** Nisha D Sherma, Chad R Borges, Olgica Trenchevska, Jason W Jarvis, Douglas S Rehder, Paul E Oran, Randall W Nelson, Dobrin Nedelkov

**Affiliations:** The Biodesign Institute at Arizona State University, Tempe, AZ 85287 USA; Department of Chemistry & Biochemistry at Arizona State University, Tempe, AZ 85287 USA

**Keywords:** Proteomics, MALDI-TOF, Biomarker discovery, Immunoassay, Quantification, Post-translational modifications, MIF

## Abstract

**Background:**

The cytokine MIF (Macrophage Migration Inhibitory Factor) has diverse physiological roles and is present at elevated concentrations in numerous disease states. However, its molecular heterogeneity has not been previously investigated in biological samples. Mass Spectrometric Immunoassay (MSIA) may help elucidate MIF post-translational modifications existing *in vivo* and provide additional clarity regarding its relationship to diverse pathologies.

**Results:**

In this work, we have developed and validated a fully quantitative MSIA assay for MIF, and used it in the discovery and quantification of different proteoforms of MIF in serum samples, including cysteinylated and glycated MIF. The MSIA assay had a linear range of 1.56-50 ng/mL, and exhibited good precision, linearity, and recovery characteristics. The new assay was applied to a small cohort of human serum samples, and benchmarked against an MIF ELISA assay.

**Conclusions:**

The quantitative MIF MSIA assay provides a sensitive, precise and high throughput method to delineate and quantify MIF proteoforms in biological samples.

## Background

MIF (Macrophage migration inhibitory factor) is a widely expressed 12.3 kDa cytokine with diverse physiological activities. It was first discovered in 1966 by Bloom and Bennett [1] as a component of an exudate that demonstrated the ability to inhibit random movement of macrophages in vitro. It was successfully cloned by Weiser *et al.* [2], revealing the approximate molecular weight of MIF, and facilitating preliminary investigations of its physiological activities. More recently it has been determined that MIF activities are mediated through extracellular receptor CD74 [3], with activation of the intracellular signaling cascade facilitated by CD44 [4]. MIF also functionally binds with chemokine receptors CXCR2 and CXCR4 [5], and the intracellular protein Jab1 [6]. Unlike most other cytokines, which are synthesized in a *de novo* manner, MIF is expressed constitutively and stored intracellularly [7].

In recent years our knowledge of MIF function has expanded to encompass diverse physiologic functions, including immune [7,8], neuroendocrine [9,10], and enzymatic [11–13] activities. Of note, MIF possesses several catalytic properties, including thiol protein oxidoreductase [12] and tautomerase activities [11,13].

In vitro, murine, and clinical studies have indicated that MIF is a key regulator of both adaptive and innate immune functions, and also modulates the inflammatory response [7,8]. MIF stimulates the release of other pro-inflammatory molecules via an autocrine mechanism [14], and acts in concert with corticosteroids to regulate the inflammatory response [15]. In humans, increased circulating concentrations of MIF have been reported in inflammatory conditions such as sepsis [16,17] and rheumatoid arthritis [18]. MIF has demonstrated potential to serve as a prognostic marker in critically ill sepsis patients [19], and as a marker of pulmonary function following cardiopulmonary bypass [20].

A number of in vitro, *in vivo*, and clinical studies have indicated a potential involvement of MIF in the development of cardiovascular and metabolic disorders [21–28]. It has been found that MIF might be involved in the early stages of plaque development [22], and that it can promote destabilization of atherosclerotic plaques [26,28]. In mouse models, it can promote intimal thickening [23,27] and induce cardiac dysfunction [24], although one study [29] found that MIF may exert cardioprotective effects in the context of ischemic injury. Numerous clinical studies have reported elevated circulating levels of MIF in the context of cardiovascular and metabolic pathologies, including acute myocardial infarction [21,30], chronic kidney disease [31], and type 2 diabetes [32–34].

Studies of MIF function and expression in cancer have revealed a largely pro-tumorogenic role. MIF inhibits the action of tumor suppressor p53 [35], and has been implicated in the development and progression of breast [36] and lung cancer [37,38]. MIF has also been noted to circulate at elevated levels in a number of cancer subtypes, including ovarian [39], colorectal [40], breast [36], and prostate cancer [41].

While immunoassays such as ELISA and RIA are commonly used for measuring circulating protein concentrations, mass spectrometry-based approaches are becoming increasingly relevant to the clinical setting for diagnostic applications [42–44]. Recently, Campa *et al.* developed a mass spectrometry-based platform for MIF and cyclophilin A to quantify these proteins in non-small cell lung cancer (NSCLC) [45].

In recent years, proteomic and mass spectrometry techniques have furthered the discovery and comprehension of protein post-translational modifications (PTMs), which may have unique physiologic activities, and can be associated with or be by products of disease processes [46,47]. As such, PTMs may potentially serve as clinically useful markers for diagnosis, prognoses, or therapy monitoring. Accordingly, it is imperative to develop analytical techniques that can not only quantify total protein levels in biological samples, but also have the ability to discriminate between different isoforms of the target protein.

Several in vitro studies have demonstrated unique biological activities of MIF post-translational modifications. It was recently discovered that MIF is the main target of PEITC (phenethyl isothiocyanate) which binds intracellular MIF at the *N*-terminus, diminishing MIF tautomerase enzyme activity [48]. Additionally Watarai *et al.* [49] demonstrated in vitro cysteinylated MIF (described by its synonym GIF) to possess elevated physiologic activity and specific immunosuppressive activities not observed for unmodified MIF, and determined the site of this modification.

While in vitro investigations have provided valuable insights into the activities of post translationally modified forms of MIF, studies of the heterogeneity of MIF in biological samples have not been previously performed. While total concentration of circulating MIF can be efficiently analyzed by ELISA assays, distinct forms of MIF cannot be differentiated. As such, a technique that can provide qualitative and quantitative analyses of MIF levels in biological matrices could facilitate the discovery of previously undetected MIF proteoforms, and may further our comprehension of the relationship of MIF with diverse pathologies.

Mass Spectrometric Immunoassay (MSIA), an immunoenrichment technique that was developed by Nelson *et al.* [50], utilizes antibodies immobilized to an activated solid support within an affinity pipette tip to capture the target protein species from a biological sample, which is then followed by elution and mass spectrometric analysis. Quantitative MSIA analyses can be performed wherein an internal reference standard is introduced into the biological sample and co-captured with the target protein in parallel to generate a standard curve. A number of MSIA assays have been developed for either single or multiple target proteins, with the ability to provide simultaneous qualitative and quantitative analyses within a single assay [51–53]. Here we describe the development, validation, and application of a qualitative and quantitative MSIA assay for Macrophage Migration Inhibitory Factor (MIF). The assay can both quantify and discern the molecular heterogeneity of this multifunctional cytokine.

## Results

### Assay development

Pooled human plasma from healthy donors was used for the initial assay development. It was determined experimentally that an addition of detergent buffer provided optimal MIF signal-to-noise ratio, with minimal presence of non-specifically bound proteins in the mass spectra. The detergent buffer had been developed for prior MSIA analyses [54,55].

Important to the development of the quantitative assay is the selection of an internal reference standard (IRS) that can simultaneously be retrieved from the sample along with the native protein and produces a consistent signal in the mass spectrum that is distinct from any naturally occurring isoforms of the target protein. We selected *C*-terminally labeled His tag MIF which has a mass of 13,411 Da. By selecting an internal reference standard that is in the same m/z range as human MIF, but adequately shifted from human MIF in a spectrum region free of peaks, potential overlap with endogenous forms of human MIF is averted.

**Figure 1:**
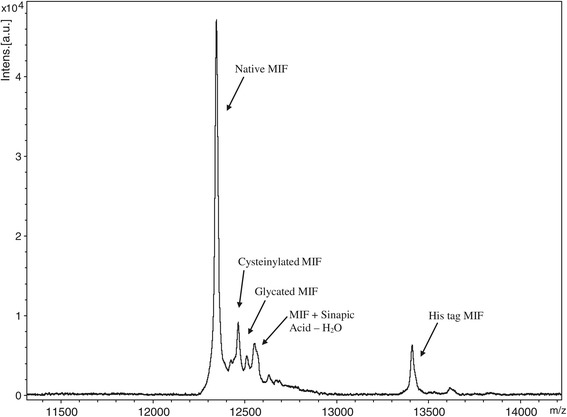
**Representative MIF Spectra from MSIA performed on healthy male serum sample with peaks corresponding to native (intact) MIF, cysteinylated MIF, glycated MIF, sinapic acid matrix adduct, and His tag MIF.**

Shown in Figure 1 is a representative mass spectrum from the MIF MSIA performed on a serum sample to which 10 ng/mL His tag MIF has been added as an internal reference standard (IRS). Peaks in the mass spectrum correspond to endogenous forms of MIF, including native (intact) MIF (m/z_obs_ = 12346.1), cysteinylated MIF (m/z_obs_ = 12465.0, m/z_calc_ = 12465.3) and glycated MIF (m/z_obs_ = 12509.6, m/z_calc_ = 12508.3). Also present are peaks of the sinapic acid matrix adduct (m/z_obs_ = 12551.9, m/z_calc_ = 12552.2) and His tag MIF (m/z_obs_ = 13411.1, m/z_calc_ = 13411.2).

Also important to assay development is the selection of a suitable matrix in which to perform standard curve dilutions. We considered animal plasma due to similarity in properties to human plasma. However, this was not feasible, due to the cross reactivity of the MIF antibody with endogenous goat, bovine, and equine MIF. As such, we utilized 10 mg/mL of Human Serum Albumin in PBS, a solution roughly iso-tonic and iso-osmotic to human plasma/serum.

### Assay validation

Recent years have brought about rapid advances in bioanalytical methodologies, and an increase in the routine research [47] and clinical use of mass spectrometers [42,43] for quantitative analyses. As such, there is a growing interest in streamlining pre-study validation measures to ensure reliable assay performance [56–59]. The performance characteristics that were evaluated for the MIF MSIA assay included intra-assay (within run) and inter-assay (between runs) precision, spiking recovery, and dilution linearity.

**Figure 2:**
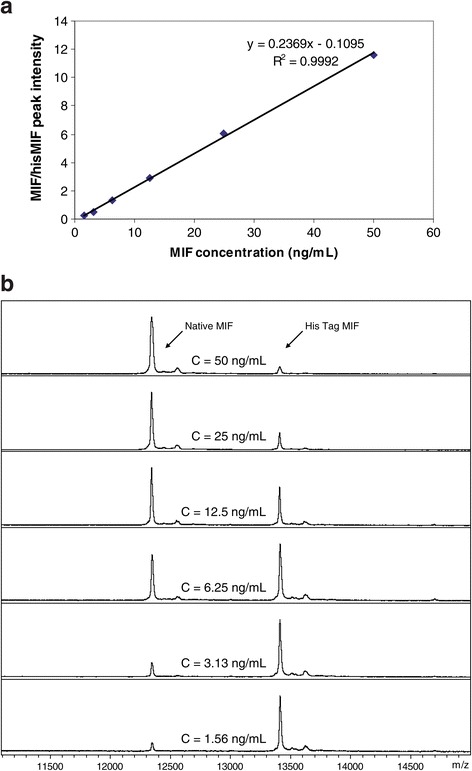
**Representative standard curve for MIF MSIA with linear range of 1.56 - 50 ng/mL. a)** MIF-to-His tag MIF ratios are plotted against concentrations of standards to create the plot; **b)** Representative MALDI TOF mass spectra from the MIF and His tag MIF standards used to generate the standard curve.

To analyze the intra-assay and inter-assay precision, a 3-day experiment was performed in which 3 replicates of a single serum sample were analyzed per day by MSIA. A standard curve was run with each analysis. A representative standard curve, along with the MIF standards mass spectra, is shown in Figure 2. A linear dynamic range was observed with the standard curve, with an average R-squared for all three days of 0.99. The intra-day CV values were 3.97%, 8.42%, and 3.14% respectively, and the inter-day CV was 8.21% (Table 1).

**Table 1:** **Intra- and inter-assay precision**

**Intra-assay CVs**				**Inter-assay CV**	
**Day:**	**1**	**2**	**3**		
**STDEVP:**	1.23	3.01	1.18	**STDEVP**	2.85
**MEAN (ng/mL):**	30.9	35.7	37.7	**MEAN**	34.8
**CV (%):**	3.97	8.42	3.14	**CV**	8.21

3 replicates of a serum sample were analyzed per day on 3 consecutive days.

To analyze the dilution linearity of endogenous MIF, two experiments involving serial two-fold dilutions were performed with serum samples with a known intact MIF concentration, on two separate days. The serum sample was diluted 2×, 4×, and 8× in a 10 ng/mL solution of HSA in PBS. The concentrations were calculated via the accompanying standard curve. The percent recovery was determined by dividing the observed by the expected concentrations (Table 2).

**Table 2:** **Assay linearity**

**Sample**	**Dilution**	**Observed**	**Expected**	**Recovery**
		**ng/mL**	**ng/mL**	**O/E %**
**1**		31.5		
	2×	15.5	15.7	98.3
	4×	7.36	7.87	93.5
	8×	4.20	3.93	107
**2**		29.2		
	2×	13.6	14.6	93.2
	4×	7.00	7.30	95.9
	8×	4.20	3.65	115

Serum samples were, on two separate days, serially diluted 2×, 4× and 8× and MSIA was performed. Intact MIF concentrations were determined from accompanying standard curve.

To analyze the spiking recovery, two experiments were performed, on two separate days, in which a serum sample containing a low endogenous concentration of MIF was fortified with recombinant MIF. For each experiment, four 200 μL aliquots of the serum sample were supplemented with 20, 10, 5, and 0 ng/mL of recombinant MIF protein respectively, via addition of 100 μL of 40 ng/mL, 20 ng/mL, 10 ng/mL, and 0 ng/mL of recombinant MIF protein (prepared in PBS-HSA buffer). A 100 μL aliquot of IRS (40 ng/mL His tag MIF in PBS-HSA buffer), and a 100 μL aliquot of detergent buffer were then added to the samples and standards, and MSIA was performed. The MIF concentrations were calculated from the accompanying standard curve equation. The percent recovery of MIF in each sample was determined from the ratio of observed to expected concentrations (Table 3).

**Table 3:** **Spiking recovery**

**Sample**	**MIF added**	**Observed**	**Expected**	**Recovery**
	**ng/mL**	**ng/mL**	**ng/mL**	**O/E%**
**1**	0	12.5		
	5	16.3	17.5	93.1
	10	20.9	22.5	92.9
	20	31.5	32.5	96.9
**2**	0	15.4		
	5	21.9	20.4	108
	10	24.8	25.4	97.7
	20	35.1	35.4	99.2

A serum sample was fortified with 0 ng/mL, 5 ng/mL, 10 ng/mL, and 20 ng/mL recombinant MIF protein and MSIA was performed. Intact MIF concentrations were determined from accompanying standard curve.

### Method comparison - MSIA and ELISA analyses of MIF concentrations in healthy serum samples

The optimized and validated MSIA MIF assay was used to analyze serum samples obtained from 22 healthy males ranging in age from 46 to 73. The serum samples were analyzed by quantitative MSIA in parallel with an accompanying standard curve, as described in the previous section. The standard curve exhibited good linearity, with an R-squared value of 0.99. A peak corresponding to glycated MIF was observed in 21 of the 22 samples, and a peak corresponding to cysteinylated MIF was observed in 18 of the 22 samples. The total MIF concentrations (sum of all MIF isoform concentrations) ranged from 11.9 to 92.7 ng/mL, with a mean of 36.2 ng/mL. Cysteinylated MIF concentrations ranged from 1.82 to 5.44 ng/mL and glycated MIF concentrations ranged from 1.82 to 17.40 ng/mL.

ELISA for the 22 serum samples was performed with Quantikine ELISA kit (R&D systems), but using Fitzgerald MIF protein in place of R&D Systems MIF standard. The samples were run in duplicate and the concentrations were averaged. The assay was performed according to the manufacturer instructions, on a Thermo Scientific Multiskan GO spectrophotometer. The MIF concentrations ranged from 11.7 to 91.7 ng/mL, with a mean of 42.8 ng/mL.

**Figure 3:**
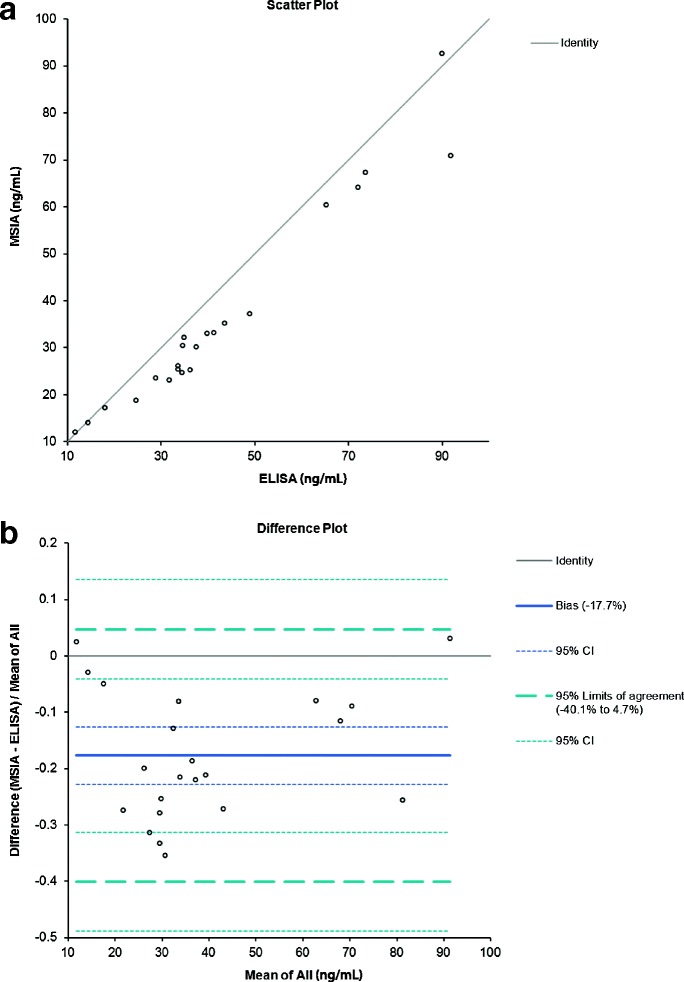
**Histogram of MIF concentrations determined by MSIA and ELISA for 22 healthy male serum samples. a)** Scatter plot showing the direct comparison between the MIF concentrations obtained with developed MSIA and reference ELISA method; **b)** Altman-Bland difference plot reveals slight negative correlation (bias = −17.7%) between the MIF concentrations obtained by MSIA vs the reference ELISA method.

A good correlation was observed between ELISA MIF and MSIA total MIF concentrations, with a negative 17.7 percent bias, as revealed by the Altman-Bland [60,61] plot (Figure 3), which shows (a) a Scatter plot and (b) a Difference plot. A Passing Bablok [62] regression equation of y = −2.76 + 0.89× was obtained, with a Cusum linearity p value of 0.78.

### Stability analyses of MIF protein standard

Changes in protein stability as a result of variations in sample storage and handling may introduce unanticipated analytical variability [63]. As such, efforts must be undertaken to experimentally identify potential sources of variability that may affect the analysis, and to establish the optimal storage and handling conditions for the samples and protein standards. Accordingly, we performed experiments to assess the stability of the 1 mg/mL recombinant MIF protein under the following conditions: storage undiluted at −80°C, storage diluted in 40 ng/mL PBS-HSA buffer at −80°C, and prolonged storage undiluted at 4°C.

To evaluate the stability of the 1 mg/mL Fitzgerald MIF protein when it is stored at −80°C but thawed and re-frozen after each use, we performed a three-day experiment in which a tube of 1 mg/mL recombinant MIF protein was stored at −80°C. On each of three days, the tube was thawed and an aliquot was removed and used to fortify a plasma sample with 20 ng/mL MIF. The tube was then placed back at −80°C. Plasma from the same individual was used on each day. Pre-aliquoted His tag MIF was added fresh on each day into the spiked plasma sample at 10 ng/mL prior to MSIA analysis. The ratio of MIF/HisMIF peak intensities decreased on each subsequent day, from 24.56 to 20.45 to 13.71, an average day-to-day decrease of 24.85%.

To determine whether the MIF protein is stable in terms of its immunoreactivity toward anti-MIF antibody when stored frozen in a solution of albumin in PBS buffer, we performed an experiment in which 1 mg/mL recombinant MIF protein was diluted to 0.1 mg/mL in 40 ng/ml PBS-HSA buffer and stored at −80°C. On each of 3 days, the 10× diluted MIF protein was taken out and used to fortify a plasma sample to 20 ng/mL. Pre-aliquoted His tag MIF was added fresh on each day into the sample at 10 ng/mL immediately prior to MSIA analysis. The MIF/HisMIF peak intensity ratios of 15.27, 15.87, and 15.20 were relatively consistent from days one through three (an average variation of 4.11% per day), indicating that the MIF protein was stable when stored frozen at -80°C.

To analyze the stability of MIF protein when stored at 4°C, we performed an experiment in which recombinant MIF protein from a freshly received vial at 1 mg/mL concentration was stored at 4°C and used to fortify a plasma sample with 20 ng/mL of MIF. The experiment was repeated after 6 and 12 days. Pre-aliquoted His tag MIF at 10 ng/mL was added fresh on each day just prior to MSIA analysis. The MIF/HisMIF peak intensity ratios were evaluated and remained relatively unchanged after both 6-day and 12-day intervals. The ratios were 15.72, 15.10, and 15.61 on days 0, 6, and 12, an average variation of 3.66% per day. As such, we concluded that the recombinant MIF protein is stable for at least 12 days stored at 4°C, and employed this sample storage for the analyses.

### Quantitative comparison of MIF in different specimen collection types

Anti-coagulants such as EDTA, heparin, and citrate are often added to plasma collection tubes, inhibiting coagulation via unique mechanisms [64]. However, differences in plasma collection tube additives may produce sample characteristics that result in differences in measurable protein concentrations, and this may introduce analytical bias into a quantitative biomarker investigation when not accounted for [63]. Hence, it is important to determine how differences in sample collection tubes may affect the analysis, and to determine the optimal specimen type for the analysis.

With this in mind, we performed a quantitative MIF MSIA analysis in order to compare the differences in measurable MIF concentrations among different sample collection types. We analyzed matched sets obtained from Bioreclamation, of 5 samples each, from 6 donors (30 samples total). Each set of 5 samples consisted of one serum sample and four plasma samples: 3.8% NaCitrate plasma, K_2_EDTA plasma, K_3_EDTA plasma, and Na_2_EDTA plasma.

**Figure 4:**
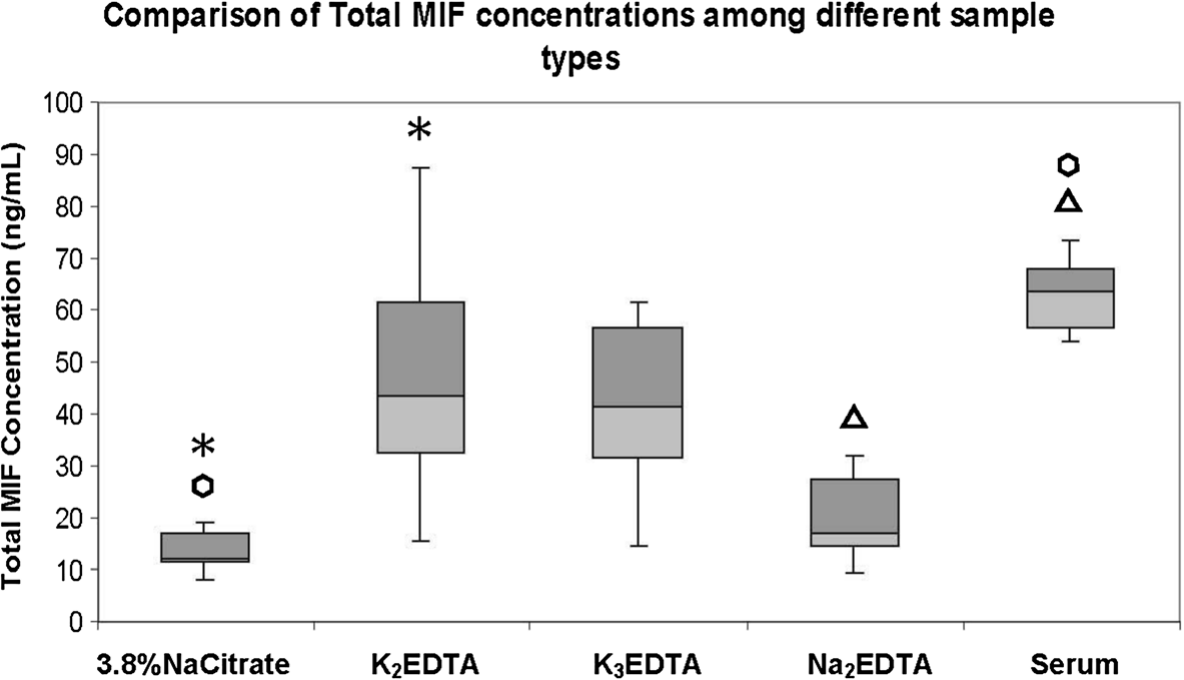
**Comparison of total MIF concentrations among different sample collection types.** Significant differences, determined by Friedman’s test with multiple pairwise comparisons, are indicated by p-values of <0.005. Matching symbols designate significant differences.

Total MIF concentrations were higher in serum than any of the plasma types, and lowest in Na_2_EDTA plasma and 3.8% NaCitrate plasma. Box Plots of total MIF concentrations comparing different sample collection types are shown in Figure 4. Friedman’s test with multiple pairwise comparisons was carried out to look for significant differences between sample types. Significant differences, indicated by p-values of <0.005, were found for serum vs 3.8% NaCitrate plasma, for serum vs Na_2_EDTA plasma, and between 3.8% NaCitrate plasma vs K_2_EDTA plasma.

### MIF glycation mapping

**Figure 5:**
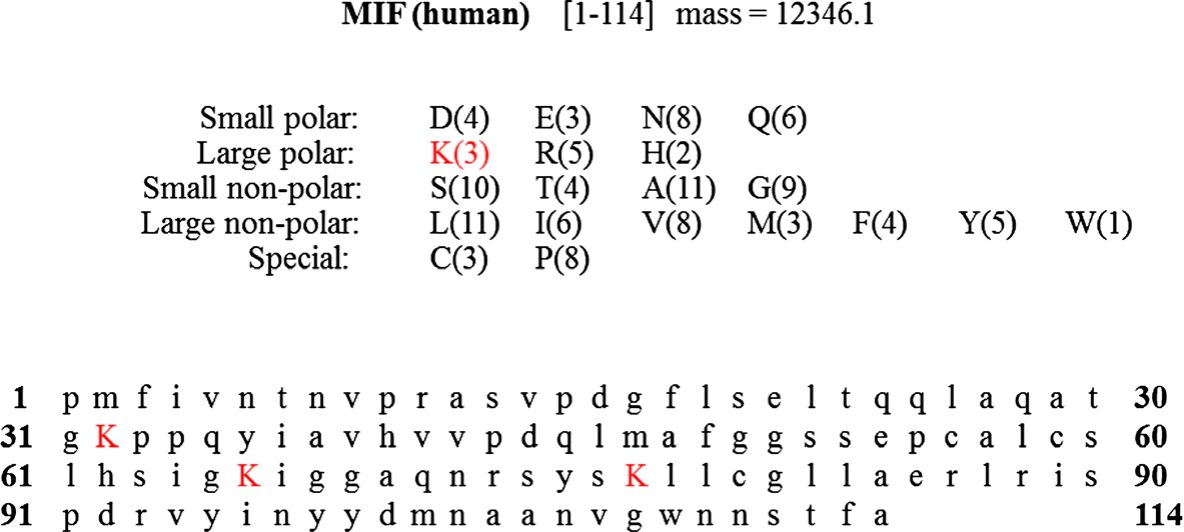
**Sequence of intact MIF, with lysine residues highlighted.**

We observed a peak corresponding to a tentative MIF glycation event in 21 of the 22 healthy male serum samples that we analysed by MSIA. To characterize this modification and potentially pinpoint its molecular location, we developed an in-solution pepsin digest method and initially applied it to in vitro glycated recombinant MIF, with the goal of applying the pepsin digestion procedure to a MSIA MIF elution of a serum sample that contains endogenous glycated MIF. MIF has 3 lysines (potential glycation sites) at positions 32, 66, and 77 - Figure 5. In-solution pepsin digest was performed for both unmodified and artificially glycated MIF as described in the methods section. In-solution pepsin digest resulted in 100% sequence coverage based on mass mapping. In the digest spectra of glycated recombinant MIF, 4 unique glycated fragments were identified on the basis of mass mapping to within 0.1 Da mass accuracy, and absence in the digest spectra of unmodified MIF, including a peptide corresponding to glycated I[4-46]L, a peptide corresponding to C[59–82]L and peptides corresponding to both single and double glycation of fragment C[59–83]L which has lysines at 66 and 77. As such, it is likely that recombinant, artificially glycated MIF can be glycated at all three lysine residues. Additional studies will need to be undertaken to determine if endogenous MIF is glycated in a site-specific manner.

## Discussion

The MSIA workflow enables an efficient and high-throughput analysis of up to 96 samples in parallel, using an automated robotic workflow for both antibody coupling and immunoaffinity enrichment of analytes. The ability to identify non-specifically bound proteins, as well as discern between various isoforms of the target protein, provides the specificity needed for clinical analyses, while the use of standard curve and an internal reference standard facilitates simultaneous analyte quantitation.

Our MSIA analysis of MIF has revealed unique qualitative and quantitative profiles of MIF within individual serum samples. Importantly, our work has facilitated the discovery of variants of MIF that cannot be individually discerned using conventional non-MS based immunoassay approaches. With the MSIA assay, we detected several isoforms of MIF to be present endogenously within serum samples, including cysteinylated and glycated MIF. To our knowledge, these forms of MIF have not previously been detected or quantified from biological samples.

Of clinical relevance to metabolic disorders is protein glycation, a non-enzymatic binding of glucose to proteins [65]. Recent studies involving in vitro glycation and subsequent in-solution proteolysis have enabled the elucidation of glycation sites of recombinant proteins, including HSA [66]. Given that glycation of MIF has not, to our knowledge, been described previously, we pursued further characterization of this modification, and determined that MIF glycation can occur at any of the three lysine residues.

Differences in measured concentrations between sample collection types, as we have observed for MIF, may be a result of multiple factors. Various anticoagulants differ in mechanisms [64], and certain additives may form complexes with serum/plasma proteins, decreasing immunoreactivity [67]. Such differences have been reported in various studies [67,68], Notably, Skogstrand *et al.* [69], in a study of inflammatory markers, concluded that higher measurable concentrations in serum compared to plasma is due to the longer storage time for serum prior to centrifugation which allows, during the coagulation process, for the gradual release of inflammatory markers that had been sequestered by blood proteins they are associated with. While discrepancies in quantitative measurements between different sample collection types are somewhat unsurprising, our results suggest that quantitative comparisons between sample cohorts should only be made amongst specimens of the same collection type.

A small negative bias of −17.7% was observed between the MSIA and ELISA MIF analyses. The reason for the bias is not clear, but interference from non-specific interactions with the antibodies employed in the ELISA analysis could play a role, resulting in elevated ELISA measured concentrations of MIF compared to MSIA.

Biomarker assays have a long history in clinical use for determining prognoses, assessment of response to therapy, and in diagnostics [44,59], and recent years have seen the emergence of diverse MS based quantitative assay methodologies. When developing a new quantitative method for biomarker analysis, assay validation is critical to ensuring a robust and reproducible method. However the criteria used to validate assays can differ, and streamlining of validation criteria is needed to ensure reliable and reproducible results. CLIA has put forth requirements for clinical labs [70], and as proteomics research has brought about novel techniques for quantitative protein analysis, interest has recently emerged in the research community with regard to harmonizing criteria for assay performance parameters. This can help enable technology transfer. Though methodologies can vary greatly, Lee *et al.* [59] have suggested a “fit-for-purpose” approach that can be applied to a wide range of analytical platforms; a set of recommended validation parameters that can be tailored to the specific needs and intended utility of the study.

In the development of our MIF quantitative MSIA assay, we have employed several of these validation measures, including intra- and inter-day precision, recovery, and dilution linearity. In addition, we have also performed assessment of protein stability, comparison with another existing assay, and comparison of different blood collection tubes. The inter-assay and intra-assay CVs and the assay linearity and spiking recovery experiments confirm the performance of the MIF MSIA assay.

In combining selective immunoaffinity enrichment of protein analytes with mass spectrometry, detailed molecular analysis is enabled, with the ability to both identify and quantify different proteoforms. With this added dimension of molecular heterogeneity, the MSIA assay provides the unique capability to address the molecular differences that may be present in diseased individuals relative to healthy subjects. Post translational modifications (PTMs) of a protein may impart unique physiological activities, and may be present in different proportions relative to the intact protein in the case of disease states. Accordingly, a targeted assay that examines the diversity of proteoforms in sample populations can facilitate the identification of unique disease biomarkers based on protein PTMs, potentially enabling more sensitive and specific detection of disease.

The molecular heterogeneity that we have identified is particularly interesting in light of the complex, multifaceted nature of this protein. A cross sectional MSIA study consisting of diverse diseased and non-disease patient samples may help elucidate the relationship between the abundance of intact, cysteinylated, and glycated MIF and the pathologies in which MIF may potentially play a role. As such, our assay may be valuable for researchers investigating the relationship between MIF and inflammatory disease states.

## Materials and methods

### Reagents

Anti-human MIF antibody was obtained from AbD Serotec (Raleigh, NC). Recombinant Human MIF protein was obtained from Fitzgerald (Acton, MA). Recombinant *C*-terminal His tag MIF was purchased from Cell Sciences (Canton, MA). *N*-methyl pyrrolidone (NMP) was obtained from Fisher Scientific (Waltham, MA). Phosphate buffered saline (PBS) and MES were obtained from Thermo Scientific (Waltham, MA). HBS-N buffer (0.1 M HEPES, 1.5 M NaCl) was prepared with NaCl and HEPES from Sigma-Aldrich (St. Louis, MO). CDI (carbonyldiimidazole), sinapic acid, ethanolamine, human serum albumin (HSA), *n*-octyl glucopyranoside, tween-20, ammonium acetate, D-(+)-glucose, diethylenetriaminepentaacetic acid (DTPA), sodium azide, toluene, tris(2-carboxyethyl)phosphine (TCEP), acetonitrile, and trifluoroacetic acid were obtained from Sigma-Aldrich (St. Louis, MO).

### Preparation of anti-MIF MSIA affinity pipettes

MSIA affinity pipette tips with porous microcolumns were obtained from Thermo Fisher Scientific (Tempe, AZ). Using a Beckman Multimek Automated 96-channel robot, the MSIA affinity pipette tips were CDI-activated, as described in previous protocols [71]. To the activated MSIA pipettes, anti-MIF antibody from AbD Serotec was covalently immobilized. It was empirically determined that 2.5 μg of antibody diluted in MES to a volume of 30 μL per well provided the optimal trade-off between antibody cost and assay performance. The antibody-derivatized MSIA tips were stored at 4°C until later use.

### Preparation of standard curve and analytical samples

We developed a 6-point standard curve to enable MIF quantification in biological samples. Prior to analysis, samples were thawed on ice, and centrifuged at 12,700 g for 5 minutes. A standard curve was performed in parallel with each sample run. For the assay standards, we used recombinant MIF protein from Fitzgerald. To perform the standard curve, the MIF recombinant protein was serially diluted in a solution of 10 mg/mL HSA in PBS. The concentrations of the MIF standards ranged from 1.56 to 50 ng/mL. His tag MIF, prepared at a concentration of 20 ng/mL in 10 ng/mL HSA in PBS, was used as an internal reference standard for quantitative analysis. A 200 μL aliquot of 20 ng/mL His tag MIF was added to 200 μL of samples and standards. The samples tray was then incubated at room temperature on a plate shaker at 750 RPM for 2 minutes to mix the sample and internal standard. A 100 μL aliquot of detergent buffer (1.5 M ammonium acetate, 0.15 M N-octyl-glucopyranoside, concentrated PBS (0.67 mol/L sodium phosphate, 1 M NaCl), and 4.4% v/v tween-20) was added to all samples and standards immediately prior to MSIA extraction.

### Mass spectrometric immunoassay

Following preparation of standards and samples, immunoaffinity extraction of MIF was carried out on a Beckman Multimek 96-channel robotic workstation, with MSIA affinity pipettes derivatized with AbD Serotec anti-MIF antibody. The pipettes were first pre-rinsed via 10 aspiration and dispense cycles in a volume of 150 μL PBS w/0.1% Tween. Next, 1000 aspiration and dispense cycles in the samples (500 μL total sample volume) were performed to allow for the flow of sample through the microcolumns and enable extraction of MIF from the samples. Next, the MSIA pipettes were rinsed sequentially with PBS w/0.1% tween (75 aspiration and dispense cycles, 150 μL volume), H_2_O (20 cycles, 150 μL), 100 mM TrisHCl (10 cycles, 150 μL) and H_2_O (30 cycles, 150 μL). Elution of captured protein onto the MALDI target was achieved via aspiration of 6 μL of sinapic acid matrix (a saturated aqueous solution containing 33% v/v acetonitrile and 0.4% v/v trifluoroacetic acid) followed by 28 aspirate and dispense cycles and subsequent release of the droplets onto the MALDI target. The droplets were air-dried to allow for co-crystallization of the matrix and proteins. Mass spectra were acquired on a Bruker Ultraflex MALDI-TOF mass spectrometer operating in positive-ion, delayed extraction linear mode, with ion source 1 at 25.00 kV, ion source 2 at 23.10 kV, lens at 9.00 kV, 90 ns delay, and 1 GS/s sample rate. Approximately 5,000 laser shots were acquired for each mass spectrum and summed. Prior to acquisition of the mass spectra, the target mass range was externally calibrated using a mixture of calibrants obtained from Bruker Daltonics (Billerica, MA), consisting of insulin, ubiquitin, cytochrome C and myoglobin. The calibrant mix was diluted 15-fold in sinapic acid matrix and 1 μL spotted onto the MALDI target. Following data collection, the mass spectra was imported into Bruker Daltonics FlexAnalysis software, smoothed (Savitzky-Golay algorithm) and baseline subtracted (Tophat algorithm). All MIF peak intensities are entered into an Excel spreadsheet. The ratio of peak intensities of MIF standards to His tag MIF was plotted against the concentrations of the MIF standards to create the standard curve. The concentrations of endogenous human MIF, including intact, cysteinylated and glycated MIF, were computed using the equation from the standard curve.

### Human samples

Healthy pooled human plasma for initial assay development was purchased from PromedDx (Norton, MA). Healthy male serum samples were purchased from PromedDx (Norton, MA). The samples were de-identified and labeled with only a barcode. We utilized 22 of these samples, which ranged in age from 46 to 73 for analysis by MSIA and ELISA assays. Matched sets, consisting of 5 sample collection types for each individual were obtained from Bioreclamation (Hicksville, NY). The samples were de-identified and labeled with only a barcode. Sample types included: 3.8% NaCitrate plasma, K_2_EDTA plasma, K_3_EDTA plasma, Na_2_EDTA plasma, and serum. We analyzed 5 sample collection types from 6 individuals (30 samples total).

### In vitro glycation of MIF

To perform in vitro glycation, 1 mg/mL recombinant MIF protein was incubated with 0.5 M glucose solution in a 1:1 volume ratio. The glucose solution contained 0.2 M sodium phosphate buffer with 1 mM diethylenetriaminepentaacetic acid (DTPA) added to chelate trace amounts of transition metal ions (e.g. iron, copper, etc.) from the phosphate buffer which may otherwise catalyze unintended auto-oxidation reactions [72]. A 5% w/v sodium azide was added to the glucose-MIF solution to inhibit bacterial growth. A drop of toluene was added to the surface with a pasteur pipet to prevent sample evaporation. The solution was incubated at 37°C. After 11 days, a mixture of 40% unmodified, 42% singly glycated, and 18% doubly glycated MIF was obtained.

### In solution pepsin digest

To perform in-solution pepsin digest, 12 μL of 10 mM HCl, 2 μL of 50 mM TCEP in water, 1 μL of acetonitrile, and 1 μL of 0.2 g/L porcine pepsin were added to 10 μL recombinant MIF. Ten minutes after addition of pepsin solution (incubation at room temperature), 1 μL of the digest was diluted 5× with alpha cyano 4-hydroxy cinnaminic acid matrix solution (a saturated aqueous matrix solution with 33% v/v acetonitrile and 0.4% v/v trifluoroacetic acid) and all 5 μL were spotted on the target. The matrix-analyte droplet was air-dried. Mass spectra were acquired on a Bruker Ultraflex operating in positive-ion reflectron mode, with ion source 1 at 25.00 kV, ion source 2 at 21.90 kV, lens at 9.50 kV, 190 ns delay, and 2 GS/s sample rate. Approximately 1000 laser shots were acquired and summed for each mass spectrum. Prior to data acquisition, the target mass range was externally calibrated with a mixture of peptide calibrants obtained from Bruker Daltonics (Billerica, MA) consisting of bradykinin, angiotensin II, angiotensin I, Substance P, bombesin, ACTH (1–17), ACTH (18–39), and somatostatin. After data acquisition, the mass spectra were imported into MoverZ software. Peptides corresponding to C-terminal cleavages at Phe, Leu, Tyr, and Trp, with up to 10 missed cleavages, were identified and the mass list was exported into PAWS software (Proteometrics, LLC).

## Abbreviations

MIF: Macrophage migration inhibitory factor
MSIA: Mass spectrometric immunoassay
MALDI: Matrix-assisted laser desorption/ionization
TOF: Time-of-flight
CDI: Carbonyldiimidazole
HSA: Human serum albumin
PBS: Phosphate buffered saline
ACTH: Adrenocorticotropic hormone
TCEP: Tris(2-carboxyethyl)phosphine
DTPA: Diethylenetriaminepentaacetic acid
IRS: Internal reference standard
HisMIF: His tag MIF
ELISA: Enzyme-linked immunosorbent assay
RIA: Radioimmunoassay
EDTA: Ethylenediaminetetraacetic acid
CV: Coefficient of variation
PTM: Post translational modification
